# Evaluation of the LIAISON XL Zika Capture IgM II for the Diagnosis of Zika Virus Infections

**DOI:** 10.3390/v12010069

**Published:** 2020-01-07

**Authors:** Mayte Pérez-Olmeda, María Paz Sánchez-Seco, Ana Vázquez, Pilar Balfagón, Jesús de la Fuente, María Ángeles Murillo, Teodora Minguito, Fernando de Ory

**Affiliations:** 1 Laboratorio de Serología y Arbovirus, Centro Nacional de Microbiología, Instituto de Salud Carlos III, 28220 Majadahonda, Spain; mayteperez@isciii.es (M.P.-O.); paz.sanchez@isciii.es (M.P.S.-S.); a.vazquez@isciii.es (A.V.); pbalfagon@isciii.es (P.B.); jfuentel@isciii.es (J.d.l.F.); mariam.murillo@isciii.es (M.Á.M.); teodoraminguito@gmail.com (T.M.); 2 Centro de Investigación Biomédica en Red de Epidemiología y Salud Pública (CIBERESP), 28029 Madrid, Spain

**Keywords:** Zika virus, chemiluminescent immunoassay, enzyme immunoassay, dengue viruses, chikungunya virus

## Abstract

The aim of this study is to evaluate the performance characteristics of the LIAISON XL Zika Capture IgM II. For this purpose we tested 128 samples obtained from recent infections caused by the Zika (ZIKV; 74 samples), dengue (DENV; 10 samples), chikungunya (CHIK V; 11 samples), rubella (RUBV; 10 samples) and measles (MeV; 10 samples) viruses, as well as human parvovirus B19 (HPVB19; 13 samples). The results of the assay under evaluation are compared with those obtained from an indirect immunofluorescence (IIF) assay, and the discrepancies are resolved by considering other laboratory results (PCR and a plaque-reduction neutralization test). The LIAISON showed excellent sensitivity (100%). The specificity (91.25%) was hampered by some false-positive results in recent dengue virus, chikungunya virus, measles virus and human parvovirus B19 infections. The method evaluated is adequate, but the low specificity makes it necessary to consider the clinical and epidemiological contexts of patients, as well as other laboratory results.

## 1. Introduction

Zika virus (ZIKV) is a mosquito-transmitted virus belonging to the *Flavivirus* genus. Since its initial discovery in Uganda in 1947 [1], sporadic cases have been described in Africa and Asia. In 2007, a serious outbreak occurred on Yap Island, Micronesia [2]. In 2014, the virus reached Brazil [3], from where it expanded rapidly across the whole of South America and then beyond, affecting 89 countries throughout the world by July 2019 [4].

The virus causes an exanthematic disease, characterized by the presence of rashes with pruritus, arthralgia, headache, myalgia, fever and asthenia [5]. This clinical picture is shared with other viruses, including the dengue virus (DENV) (another member of the *Flavivirus* genus), chikungunya virus (CHIKV) and other exanthematic agents, such as the rubella (RUBV) and measles (MeV) viruses, as well as human parvovirus B19 (HPVB19) [5,6], whose clinical diagnosis is difficult. It is especially important to ensure the differential diagnosis of DENV (due to crossreactivity with members of the *Flavivirus* genus) and the alphavirus CHIKV—the three viruses are transmitted by mosquitoes of the genus *Aedes* and, consequently, have a similar geographical distributions [7].

Serological diagnosis of ZIKV is based on specific IgM detection. The first assay applied was indirect immunofluorescence (IIF) with ZIKV-infected cells. Using this approach, however, the high degree of crossreactivity between ZIKV and other flaviviruses makes correct serological diagnosis difficult. ZIKV non-structural (NS) protein 1 has been identified as being largely specific to the virus [8], prompting new assays to be developed. In recent years, some alternative approaches have been used, above all enzyme and chemiluminescent immunoassays (ELISA and CLIA, respectively). The aim of the study reported in this paper was to compare the performance of the CLIA LIAISON XL Zika Capture IgM II (Diasorin, Italy) method for the diagnosis of ZIKV infection against that of an IIF assay.

## 2. Materials and Methods

### 2.1. Samples

A total of 128 samples from 123 patients were analyzed. They were grouped as follows:

- ZIKV infection (74 samples, 69 patients who reported recent travel within an endemic ZIKV area in 2016–2017). This panel included:
  ○ 46 samples from 42 cases showing ZIKV-positive IgM by IIF (42 positive, 1 indeterminate and 3 negative samples); 12 cases gave a positive result with PCR in serum (3), serum and urine (1) or urine (8).
  ○ 28 samples from 27 cases showed negative IgM, but were positive with PCR in serum (8), in serum and urine (5) and in urine (14).
- DENV infection (10 samples from 10 cases), occurring in travelers to endemic dengue regions in 2018–2019. This panel included:
  ○ Two samples from two cases with DENV-positive IgM and IgG and NS1 antigen (2 cases) and 1 sample from 1 case with positive IgM and IgG and PCR,
  ○ One sample (from 1 case) with positive IgM but a negative IgG result, and 6 samples (from 6 cases) with positive IgM and IgG. Two samples showed positive IgM against ZIKV, and the other 8 were negative.
- CHIKV infection (11 samples from 11 cases in travelers to endemic chikungunya regions in 2016–2018). All of them were diagnosed by the presence of IgG and IgM against the virus. All were IgM-negative for ZIKV by IIF.
- RUBV infection (10 samples from 10 cases) collected during an outbreak of rubella that occurred in Spain in 1996. All the samples were diagnosed by specific IgM against the virus; IIF showed all of them to be IgM-negative for ZIKV.
- MeV infection (10 samples from 10 cases) collected during an outbreak of measles that occurred in Spain in 1991. All the samples were diagnosed by specific IgM against the virus; IIF showed all of them to be IgM-negative for ZIKV.
- HPVB19 infection (13 samples from 13 cases) collected in 2007–2009 of recent infection with HPVB19. The samples were diagnosed by specific IgM against the virus; all were IgM-negative for ZIKV by IIF. Four samples gave a positive result with PCR for HPVB19.

All samples were stored at −20 °C until used in this study.

The study was approved by the Ethics Committee of the Institute of Health Carlos III. (Code: CEI PI 16_2019-v2, 30 December 2019)

### 2.2. Methods

IgM and IgG were assessed for ZIKV and CHIK with IIF, using commercial assays (Euroimmun, Lübeck, Germany) diluted 1:10; levels of IgM were determined after removing IgG from the sample (RF Absorbens, Siemens, Marburg, Germany). DENV IgM was tested by capture ELISA (Panbio, Standard Diagnostics Inc, Geonggi-do, South Korea) using a previously described antigen control assay [9]. RUBV and MeV IgM were analyzed by indirect ELISA (Enzygnost, Siemens), and HPVB19 IgM by capture ELISA (Biotrin-Diasorin, Dublin, Ireland). DENV NS1 antigen was determined by ELISA (Platelia NS1, Bio-Rad, Marnes-La-Coquette, France). ZIKV RNA was detected with the RealStar Zika Virus assay (altona Diagnostics, Hamburg, Germany). Finally, the ZIKV plaque reduction neutralization test (PRNT) was carried out with an in-house method using Vero E6 cells (ATCC CRL-1586, American Type Cell Collection, Manassas, Virginia) and 100 TCID50 of ZIKV (African strain MR-766, isolated from a rhesus monkey in the Zika Forest, Uganda, in 1947, and maintained in Vero E6 cells). For neutralizing antibodies, samples were tested using two-fold dilutions starting from 1:8. Samples were considered positive if neutralization of viral growth at a dilution >1:128 was observed; samples with titres between 1:8 and 1:128 were considered indeterminate; and samples with a titre <1:8 were considered negative. Some samples were tested by ELISA for IgG ZIKV by using a commercial assay using NS1 as antigen (Euroimmun).

All samples were tested by the LIAISON XL Zika Capture IgM II (Diasorin, Saluggia, Italy) on the LIAISON XL Analyzer platform (Diasorin). The assay included specific IgM and IgG determinations (the latter being used as an aid in interpretation) against ZIKV based on CLIA capture methodology. Both determinations used the NS1 protein of ZIKV as an antigen. The results were interpreted as indicated in Table 1.

## 3. Results

Overall, analyzing the samples by IIF for IgM against ZIKV showed that 44 samples were positive (42 of the ZIKV cases and 2 of the DENV cases), 1 was indeterminate (a case of ZIKV) and 83 were negative (31 of the cases with suspected ZIKV infection. Eight of the 10 cases infected with DENV and all of the samples from CHIKV, MeV, RUBV and HPVB19 infections were negative via IIF.

When analyzing the 74 samples from recent ZIKV infection (panel i), a positive result was obtained by LIAISON in 47 (34 of the 42 IIF-positive samples, showing an indeterminate result with IIF, and 12 out of 31 IIF-negative samples); the other 27 samples ended in a negative result.

On the other hand, when the samples from infections other than ZIKV were tested, a positive result was obtained in 1/10 DENV (panel ii), 1/11 CHIKV (panel iii), 0/10 RUBV (panel iv), 1/10 MeV (panel v) and 4/13 HPVB19 (panel vi) infection samples. 

Thus, a positive result was obtained with LIAISON XL Zika Capture IgM in 54 samples, and a negative result was found in 74 samples.

A comparison of the results obtained with the LIAISON XL Zika Capture IgM with those obtained by IIF is summarized in Table 2. An agreement of 76.6% (98/128) was obtained.

Table 3 lists the 30 samples with discrepant results. In samples 1–13, the positive results with the LIAISON XL Zika Capture IgM were classified as true positives, since they came from cases with a positive PCR result in serum (samples 3–7) or in urine (samples 1, 2 and 8–13). Sample 14 was considered “unclassifiable”, since, being discrepant in IIF and LIAISON, it was the only indeterminate result in PRNT. Samples 15–21 were considered true negatives since they gave a negative result in PRNT for ZIKV (samples 15–18) or because they were IgG-negative with ELISA, even though they were positive with IIF (samples 19–21). Samples 22 and 23 came from a case of dengue, with a positive PCR in previous samples, so they were considered true negatives. Finally, samples 24–30 were considered false positives, since they came from cases of DENV (24), CHIKV (25), MeV (26) and HPVB19 (27–30, including a sample positive for HPVB19 PCR).

Table 4 compares the results of LIAISON against the reference criteria after the classification of the discrepant samples, excluding the one finally considered as unclassifiable. Agreement, sensitivity and specificity values were 94.5%, 100% and 91.25%, respectively.

## 4. Discussion

The laboratory diagnosis of ZIKV infections is based primarily on the detection of specific IgM. The methods used are mainly IIF, using virus-infected cells and ELISA and CLIA, which use specific proteins, primarily NS1, which is recognized as being largely specific to the virus [8]. ZIKV-specific antibodies can yield false-positive results when tested. These mainly arise from crossreactions with other flaviviruses and are of particular significance when detecting ZIKV IgM. DENV is the most important of these for establishing a differential diagnosis, since, apart from their similar clinical symptomatology, the two viruses share epidemiological and vector-transmission characteristics. Obtaining false-positive results is of particular relevance when using IIF assays, which use cells infected with the virus as the antigen. The specificity of IgM detection improves when virus-specific antigens are applied. Thus, when ELISA assays using the NS1 protein are compared with IIF—and other laboratory results are used for the final classification of cases, such as those from PCR or PRNT—the concordance, sensitivity and specificity values of the first assay are 81.1%, 65.9% and 100%, with IIF being 86.0%, 96.8% and 72.5%, respectively, which represents a substantial improvement in specificity [11]. Assays with the NS1 protein show greater specificity than those obtained using other virus proteins such as E/prM recombinant protein, although sensitivity may be affected [12].

The LIAISON methodology is applicable to the serological diagnosis of bacteria, viruses and parasites, and shows good performance characteristics. Its application to the diagnosis of ZIKV infection has been evaluated in this study. An earlier version of the LIAISON assay yielded a sensitivity of 85% in cases confirmed by PRNT, with the value decreasing when samples with a positive PCR result were included since they were taken at acute stages of the disease [13]. This observation has been made in other studies conducted with ELISA-NS1 [14,15], and suggests that the production of IgM versus NS1 could be delayed, and thereby not detected in the early acute stage (<5 days after the onset of symptoms). However, another study gave positive results with LIAISON in 12/17 samples (70.6%) collected within the first five days of the disease [16]. In this sense, in the present study, 13 samples with suspected ZIKV infection with a negative or undetermined IgM result for IIF and a positive result with LIAISON gave positive results in serum PCR (5 cases) or urine (8 cases), suggesting that these samples were taken at very early stages (samples 1–13, Table 3). Additional studies are needed to elucidate the kinetics of IgM production with the assay under evaluation by studying follow-up samples of ZIKV recent infections.

An important aspect in the validation of tests consists of identifying reactivities that allow the specificity to be assessed. For this reason, it is important to select samples of cases in which problems of differential diagnosis arise. Previously, false-positive reactions have been identified in LIAISON in cases of infection by DENV, *Plasmodium falciparum*, *P. vivax* and *P. malariae*, as well as the Epstein–Barr virus and Cytomegalovirus in patients without a history of traveling to ZIKV-endemic areas [16]. In the present study, we have attempted to assess the specificity using samples of dengue (because of its crossreactivity with flavivirus) and CHIKV (because it is endemic in the same region as ZIKV and DENV), as well as RUBV and MeV (given the current importance of surveillance programs that aim to eliminate these diseases) and HPVB19. Differential diagnosis problems arise with all these viruses. With regard to DENV, the assay evaluated here showed reasonable specificity, since it detects 1/10 false-positive cases, which falls within the range of previously published values (with IIF, two samples tested positive for ZIKV IgM) [16]. On the other hand, we cannot completely exclude the possibility that the sample with a positive IgM result in LIAISON for ZIKV and IgM against CHIKV (25) actually corresponds to a double infection with both viruses, since the sample was obtained when both viruses were simultaneously circulating (2018).

No results have been published about the heterologous reaction with RUBV, MeV and HPVB19. In the present study, positive results were detected in 1/10 cases of MeV and in 4/13 samples of HPVB19. It is important to note that the samples were taken from these patients either in 1991 (the measles cases) or between 2007 and 2009 (HPVB19), during the period before a laboratory diagnosis of ZIKV was required. These results are of interest in the context of measles and rubella elimination plans, but further studies are needed to confirm the importance of heterologous reactivities.

The present study has some limitations. The first concerns the limited clinical information available, especially for HPVB19 cases, without which it is not possible to identify potential causes of false-positive results. The second is the absence of information about patients’ travel histories in the cases of MeV and HPVB19, which gave positive results with the LIAISON method, although the samples were collected years before we were first alerted to ZIKV. Finally, the lack of information about the time elapsed in the ZIKV cases between the onset of the disease and the collection of the sample makes it difficult to evaluate the discrepant results in the IIF and LIAISON assays.

## 5. Conclusions

In conclusion, the LIAISON XL Zika Capture IgM II method shows excellent sensitivity for identifying recent ZIKV infections. However, non-specific reactivity was obtained in some of the samples infected by agents for which a differential diagnosis must be established, such as cases of DENV, CHIKV, MeV and HPVB19. This makes it necessary for any result to be assessed in the clinical and epidemiological context of the patient.

## Figures and Tables

**Table 1:** Interpretation of results of the LIAISON XL Zika Capture IgM II [10].

ZIK-M Index	ZIK-C Index	Result	Interpretation
<1.0	Any value	Negative	No detectable levels of IgM antibodies to Zika virus were found. A negative result does not always rule out acute or recent Zika virus infection. IgM antibodies to Zika virus may not be detectable if the infection is in a very early stage, before the patient has developed Zika-specific IgM antibodies or if the infection is in a late stage of infection after the IgM antibodies have subsided.
≥1.0 to <2.2	<4.0		
	≥4.0	Presumptive Zika IgM-positive	Presence of detectable IgM antibodies to Zika virus. Confirmatory testing is recommended.
≥2.2	Any value		

**Table 2:** Comparison of results from the LIAISON XL Zika Capture IgM II versus IIF.

		IIF (128)		
		Positive (44)	Indeterminate (1)	Negative (83)
LIAISON^®^	Positive (54)	34	1	19
	Negative (74)	10		64

**Table 3:** Final resolution of samples with discrepant results.

Panel	Sample	PCR Serum/Urine	Zika-IgM IIF	Zika LIAISON	Zika-IgM (Index)	Zika-C (Index)	Zika-IgG ^1^	Zika PRNT	Other Results	Final Classification
i	1. ZV-39	Neg/Pos	IND	POS	2.77	0.586	Ind		DENV IgM: Neg; IgG: Neg	True positive
	2. ZV-14	ND/Pos	NEG	POS	11.9	2.5	Neg			True positive
	3. ZV-50	Pos/ND	NEG	POS	3.81	14.3	Pos	Pos		True positive
	4. ZV-53	Pos/ND	NEG	POS	3.28	12.3	Pos			True positive
	5. ZV-57	Pos/ND	NEG	POS	2.59	0.576	Pos			True positive
	6. ZV-58	Pos/Neg	NEG	POS	3.71	2.71	Pos			True positive
	7. ZV-60	Pos/ND	NEG	POS	2.65	0.359	Neg			True positive
	8. ZV-63	Neg/Pos	NEG	POS	4.87	0.476	Pos			True positive
	9. ZV-64	Neg/Pos	NEG	POS	>29.0	8.7	Pos	Ind		True positive
	10. ZV-65	Neg/Pos	NEG	POS	15.5	5.36	Pos		DENV IgM: Neg; IgG: Pos	True positive
	11. ZV-72	Neg/Pos	NEG	POS	5.27	21.5	Pos		DENV IgM: Pos; IgG: Pos	True positive
	12. ZV-73	Neg/Pos	NEG	POS	>29.0	1.49	Pos		DENV IgM: Pos; IgG: Pos	True positive
	13. ZV-74	Neg/Pos	NEG	POS	2.87	0.341	Pos		DENV IgM: ind; IgG: Neg	True positive
	14. ZV-21	Neg/ND	POS	NEG	0.933	2.38	Pos	Ind	DENV IgM: Neg; IgG: Pos	Unclassifiable
	15. ZV-4	Neg/ND	POS	NEG	1.61	0.33	Neg	Neg		True negative
	16. ZV-9	Neg/Neg	POS	NEG	0.767	0.383	Pos	Neg	DENV IgM: Neg; IgG: Pos	True negative
	17. ZV-10	Neg/ND	POS	NEG	1.01	1.3	Pos	Neg		True negative
	18. ZV-18	ND/ND	POS	NEG	0.875	0.36	Pos	Neg	DENV NS1 Ag: Pos; IgM: Pos; IgG: Pos	True negative
	19. ZV-25	ND/ND	POS	NEG	1.32	0.367	Pos/Neg	Ind	DENV IgM: Pos; IgG: Pos	True negative
	20. ZV-31	ND/ND	POS	NEG	1.05	0.287	Pos/Neg			True negative
	21. ZV-45	Neg/ND	POS	NEG	2.11	1.41	Pos/Neg		DENV IgM: Neg; IgG: Neg	True negative
ii	22. DENV_M_6	ND/ND	POS	NEG	1.72	0.367	ND		DENV PCR: Pos ; IgM: Pos; IgG: Pos	True negative
	23. DENV_M_9	ND/ND	POS	NEG	1.82	0.455	ND		DENV PCR: Pos ; IgM: Pos; IgG: Pos	True negative
	24. DENV_M_2	ND/ND	NEG	POS	2.62	0.346	ND		DENV NS1 Ag: Pos; IgM: Pos; IgG Pos	False positive
iii	25. CHIK_M_1	ND/ND	NEG	POS	3.11	0.327	ND		DENV IgM: Neg; IgG: Pos	False positive
v	26. MeV_M_6	ND/ND	NEG	POS	2.89	0.415	ND			False positive
vi	27. HPVB19_M_1	ND/ND	NEG	POS	8.73	1.22	ND			False positive
	28. HPVB19_M_4	ND/ND	NEG	POS	>29.0	0.752	ND			False positive
	29. HPVB19_M_8	ND/ND	NEG	POS	3.88	1.49	ND			False positive
	30. HPVB19_M_13	ND/ND	NEG	POS	10.2	0.688	ND		HPVB19 PCR: Pos	False positive

* ND: not done; ^1^ ZIKV IgG result with IIF, except when there were two results; the second is from ELISA; positive DENV by PCR in a previous sample.

**Table 4:** Comparison of LIAISON results after final classification of cases.

		Final Classification	
		Positive (47)	Negative (80)
LIAISON	Positive	47	7
	Negative	0	73
Agreement: 94.5% (120/127)			
Sensitivity: 100% (47/47)			
Specificity: 91.25% (73/80)			

## References

[1] Dick G.W., Kitchen S.F., Haddow A.J. (1952). Zika virus. I. Isolations and serological specificity. Trans. R. Soc. Trop. Med. Hyg..

[2] Duffy M.R., Chen T.H., Hancock W.T., Powers A.M., Kool J.L., Lanciotti R.S., Pretrick M., Marfel M., Holzbauer S., Dubray C. (2009). Zika virus outbreak on Yap Island, Federated States of Micronesia. N. Engl. J. Med..

[3] Faria N.R., Azevedo R.D.S.D.S., Kraemer M.U.G., Souza R., Cunha M.S., Hill S.C., Thézé J., Bonsall M.B., Bowden T.A., Rissanen I. (2016). Zika virus in the Americas: Early epidemiological and genetic findings. Science.

[4] World Health Organization Countries and Territories with Current or Previous Zika Virus Transmission by WHO Regional Office. https://www.who.int/emergencies/diseases/zika/countries-with-zika-and-vectors-table.pdf?ua=1.

[5] Guanche Garcell H., Gutiérrez García F., Ramirez Nodal M., Ruiz Lozano A., Pérez Díaz C.R., González Valdés A., Gonzalez Alvarez L. (2019). Clinical relevance of Zika symptoms in the context of a Zika Dengue epidemic. J. Infect. Public Health.

[6] Muzumdar S., Rothe M.J., Grant-Kels J.M. (2019). The rash with maculopapules and fever in adults. Clin. Dermatol..

[7] Houé V., Bonizzoni M., Failloux A.B. (2019). Endogenous non-retroviral elements in genomes of *Aedes* mosquitoes and vector competence. Emerg. Microbes Infect..

[8] Stettler K., Beltramello M., Espinosa D.A., Graham V., Cassotta A., Bianchi S., Vanzetta F., Minola A., Jaconi S., Mele F. (2016). Specificity, cross-reactivity, and function of antibodies elicited by Zika virus infection. Science.

[9] Domingo C., de Ory F., Sanz J.C., Reyes N., Gascón J., Wichmann O., Puente S., Schunk M., López-Vélez R., Ruiz J. (2009). Molecular and serologic markers of acute dengue infection in naive and flavivirus-vaccinated travelers. Diagn. Microbiol. Infect. Dis..

[10] Diasorin (2019). LIAISON^®^XL Zika Capture IgM RFE 317150, Instructions for Use.

[11] De Ory F., Sánchez-Seco M.P., Vázquez A., Montero M.D., Sulleiro E., Martínez M.J., Matas L., Merino F.J., Working Group for the Study of Zika Virus Infections (2018). Comparative evaluation of indirect immunofluorescence and NS-1-based ELISA to determine Zika virus-specific IgM. Viruses.

[12] Basile A.J., Goodman C., Horiuchi K., Sloan A., Johnson B.W., Kosoy O., Laven J., Panella A.J., Sheets I., Medina F. (2018). Multi-laboratory comparison of three commercially available Zika IgM enzyme-linked immunosorbent assays. J. Virol. Methods.

[13] Sloan A., Safronetz D., Makowski K., Barairo N., Ranadheera C., Dimitrova K., Holloway K., Mendoza E., Wood H., Drebot M. (2018). Evaluation of the Diasorin LIAISON^®^ XL Zika Capture IgM CMIA for Zika virus serological testing. Diagn. Microbiol. Infect. Dis..

[14] Steinhagen K., Probst C., Radzimski C., Schmidt-Chanasit J., Emmerich P., van Esbroeck M., Schinkel J., Grobusch M.P., Goorhuis A., Warnecke J.M. (2016). Serodiagnosis of Zika virus (ZIKV) infections by a novel NS1-based ELISA devoid of cross-reactivity with dengue virus antibodies: A multicohort study of assay performance, 2015 to 2016. Euro Surveill..

[15] Amaro F., Sánchez-Seco M.P., Vázquez A., Alves M.J., Zé-Zé L., Luz M.T., Minguito T., De La Fuente J., De Ory F. (2019). The application and interpretation of IgG avidity and IgA ELISA tests to characterize Zika virus infections. Viruses.

[16] Van der Beken Y., De Geyter D., Van Esbroeck M. (2019). Performance evaluation of the Diasorin LIAISON^®^ XL Zika capture IgM CLIA test. Diagn. Microbiol. Infect. Dis..

